# Prefabricated platinum nanomaterial matrix for MALDI-MS imaging of oligosaccharides and lipids in plant tissues

**DOI:** 10.3389/fpls.2023.1105374

**Published:** 2023-01-18

**Authors:** Yu-Lin Shen, Si-Jia Zhuang, Fan Yang, Can Gong, Xu Xu

**Affiliations:** School of Chemical and Environmental Engineering, Shanghai Institute of Technology, Shanghai, China

**Keywords:** matrix-assisted laser desorption ionization mass spectrometry imaging, matrix, platinum nanomaterials, plant tissue, oligosaccharide, lipid

## Abstract

Matrix-assisted laser desorption ionization mass spectrometry imaging (MALDI-MSI) can visualize the spatial distribution characteristics of molecules in tissues in situ, in which the matrix plays a key role. In this paper, we propose a platinum nanomaterial pre-coated matrix, which can be prepared in bulk by sputtering platinum nanoparticles onto slides using an ion sputterer and then used for MALDI-MS analysis by placing tissue sections on the matrix. We used this matrix for MALDI-MS imaging analysis of corn kernels and germinated wheat sections, and the results show that triacylglycerides were mainly distributed in the embryo of corn kernels and germinated wheat, and sugars were mainly distributed in the endosperm, with the highest content of disaccharides.It provides a simple and reliable experimental condition for analyzing the distribution of oligosaccharide and lipid components in plant tissues.

## Introduction

MALDI MSI is an efficient technique for plant metabolomics research (Susniak et al., 2020). It can be used to characterize the spatial distribution of metabolites in plants (Li et al., 2018) and reveal the spatial and temporal metabolic patterns of plant secondary metabolite production, transport, and storage (Sun et al., 2020). Conventional organic matrixes such as α-cyano-4-hydroxycinnamic acid (CHCA) and 2,5-dihydroxybenzoic acid (DHB) have the propensity to produce non-uniform crystals with analytes, maybe resulting in poor repeatability and misinterpretation of MS images (Mueller et al., 2021).

As alternatives to traditional matrixes, researchers have proposed metal/metal oxide-based (Sakurai et al., 2018; Sagandykova et al., 2022), carbon-based (Xu et al., 2021), and silicon-based (Dou et al., 2022) matrixes. Metal particles with nanoscale diameters are of interest due to their unique plasmon absorption in the visible region, in which gold nanoparticles (Au NP) and silver nanoparticles (Ag NP) are the often utilized metal matrixes. However, they are susceptible to gold and silver cluster ion. Studies have shown platinum nanoparticles (Pt NP) have softer surfaces than Au and AgNPs (Picca et al., 2017). Pt is very stable and does not oxidize quickly under ambient atmospheric conditions. Black Pt nanoparticles are effective MALDI-MS matrix for the whole UV-Vis region at laser wavelengths. Due to its extremely high melting temperature (2045 K), high crystallinity, and relatively low thermal conductivity (71.6 W m^-1^K^-1^ at 300 K), Pt can reduce fragmentation of metal nanoparticles such as clusters and is a good candidate matrix for MALDI-MS (Kawasaki et al., 2007).

In this study, we present an imaging method using platinum nanomaterials as a pre-coated matrix for visualizing and analyzing the spatial distribution of metabolites in plant tissues. This pre-coated matrix can be prepared in bulk using an ion sputterer. Tissue sections can be placed directly on the pre-coated matrix layer to conduct MALDI MSI studies, which reduces sample component diffusion, improves reproducibility, and simplifies experimental steps. The pre-coated matrix was successfully used for imaging oligosaccharide and lipid components in germinating wheat and corn sections.

## Materials and methods

### Materials

Gelatin, L-phenylalanine (99%), L-tyrosine (99%), and naringin (98%) were purchased from Adamas-beta(Shanghai,China).Palmitic acid (>97%), stearic acid (>98%), linoleic acid (>97%), and D-(+)-raffinose(>98%) were purchased from TCI Chemical Industries (Shanghai, China).Abscisic acid (ABA, 98%) were purchased from Sigma-Aldrich(St. Louis, USA).Dipalmitoyl phosphatidylcholine (DPPC) was purchased from Avanti Polar Lipids (Alabama, USA). Indolebutyric acid (99%)and α-cyclodextrin (≥98%) were purchased from Yuanye Biotechnology (Shanghai, China).Glycerol trioleate (99%) and 2,5-dihydroxybenzoic acid (DHB, 99%)were purchased from J&K Scientific (Beijing, China).Paeoniflorin (98%), sucrose (AR), and L-glutamic acid (≥99%) were purchased from Titan Technology (Shanghai, China).Ethanol (absolute, AR grade) was purchased from Sinopharm (Beijing, China).Methanol (HPLC grade) and dichloromethane (AR grade) were purchased from Boer Chemical Reagent (Shanghai, China), deionized water was obtained from Milli-Q Water Purification System (Millipore, Billerica, MA, USA).

Tissue-Tek OCT compound was purchased from Sakura Finetek (CA, USA). Microscope cover glasses (22mm × 22mm) were purchased from Citotest Scientific (Nanjing, China).We purchased fresh sweet corn from a local supermarket. Wheat seeds (Jimai 22) were from Crop Research Institute, Shandong Academy of Agricultural Sciences.

### Preparation of platinum pre-coated matrix

The matrix was prepared using commercial magnetron sputtering equipment (Hitachi E-1010, Tokyo, Japan) with high-purity platinum targets (>99.9%). The distance between the platinum target and the sample was approximately 3.5 cm during sputtering. The platinum was deposited on a glass coverslip with a 20 mA discharge current under reduced pressure (15 Pa).

### Preparation of authentic compound solutions

Water-soluble authentic compounds, including L-phenylalanine, L-tyrosine, L-glutamic acid, sucrose, raffinose, and α-cyclodextrin, were prepared in water. Water-insoluble authentic compounds, including palmitic acid, stearic acid, linoleic acid, naringin, paeoniflorin, abscisic acid, Indolebutyric acid, and glycerol trioleate, were prepared in anhydrous ethanol. DPPC was prepared in dichloromethane. All of the authentic compounds concentrations were 1 mg/ml. 2 μL solution of each authentic compound was spotted on a pre-coated platinum matrix pre-heated to 70°C to ensure uniform deposition. For comparison of platinum nanomaterials and organic matrix, 10 mg/ml of DHB was prepared in methanol-water solution (1:1, V/V) for positive ion mode analysis. 2 μl DHB was spotted on a glass coverslip to dry, followed by 2 μl of authentic compound solution.

Preparation of soybean extract: 10g of soybean were mashed and sieved, then 1 g of the sieved soybean powder was added to 50 mL of methanol and sonicated for two hours. The precipitate was centrifuged to extract the supernatant.

### MALDI MSI sample preparation

Conditions for germination: The wheat seeds were rinsed five times with deionized water. They were soaked in a beaker with water for 2 hours, then transferred to a tray lined with wet paper towels, covered with wet gauze, placed at room temperature, and shielded from light to facilitate germination. The seeds were kept moist by regular application of water to the surface.

Sample preparation: Fresh samples (corn kernels, germinated wheat) were embedded in gelatin solution (10%, W/V) and then frozen in a -80°C refrigerator. For cryosectioning, frozen samples were fixed directly on sample holders with optimal cutting temperature (OCT) compounds, and 12 μm-thick tissue sections were obtained using a slicer at -20°C and thaw-mounted on slides pre-coated with platinum matrix. To prevent condensation, tissue sections were dehydrated for 30 minutes in a vacuum desiccator at room temperature.

### MALDI MSI

MALDI MSI measurements were performed using a 7.0 T SolariX FTICR mass spectrometer (Bruker Daltonics, USA) equipped with dual ion sources (ESI and MALDI) and a Smartbeam II 355 nm laser. The m/z range obtained in positive ion mode was 53-1000. The single scan spectrum consisted of 25 cumulative 600 Hz laser shots with “Minimum” laser focus. The laser power in the positive ion mode was 70%. The spatial resolution of MALDI images of germinating wheat tissue sections was 100 μm, while the spatial resolution of MALDI images of sweet corn tissue sections was 150 μm. MALDI MS data processing was performed with Bruker DataAnalysis 4.0.HyStar software (Version 3.4) was used to capture imaging data, and Bruker FlexAnalysis software was used to handle the imaging data.

## Results and discussion

### Selection of experimental conditions for pre-coated matrix

**Figure 1:**
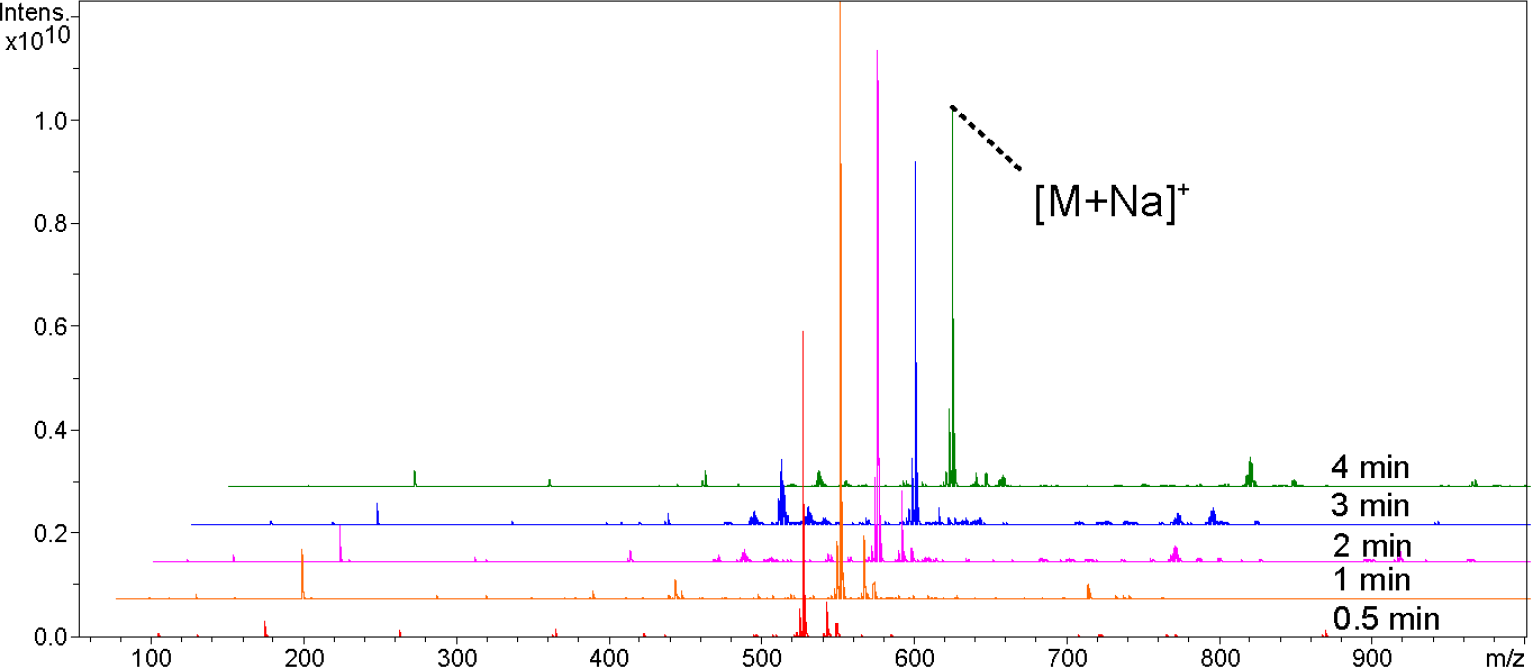
Comparison of ion signal intensities of Raffinose at 0.5, 1, 2, 3, and 4 min sputtering times.

In order to optimize the sputtering time of Pt nanomaterials, we analyzed L-phenylalanine, palmitic acid, naringin, abscisic acid, raffinose, glycerol trioleate, and DPPC in positive ion mode, and repeated them 3 times respectively. We compared the variations in their peak intensities at 0.5, 1, 2, 3, and 4 min sputtering times (**Figures 1**, **Figure S1**). With the increase in sputtering time, the peak intensities first increased and then leveled off or decreased. **Figure 1** shows the mass spectra of raffinose detected at different sputtering times; it has the highest signal intensity and less background signal when sputtering for 1 min. All following experiments utilized 1 min sputtering time.

### Evaluation of repeatability of platinum nano pre-coated matrix

Palmitic acid, raffinose, abscisic acid, and glycerol trioleate were selected to examine the intra- and inter-spot reproducibility of the platinum pre-coated matrix. MALDI MS analysis was performed on nine sample spots on one pre-coated matrix layer and nine positions on one sample spot. As shown in **Figure S2**, the intra-spot repeatability was <5%, and inter-spot repeatability was <6%, indicating that the sputtering coated Pt matrix was homogenous and provided the high repeatability for MALDI-MS of the compounds.

### Pt pre-coated matrixes for the detection of various small molecules

The detection coverage capability of the platinum pre-coated matrix was further explored in MALDI MS analysis of various plant-associated small molecules. **Figure S3** and **Table S1** show the 15 small molecule compounds detected by DHB and platinum pre-coated matrix. The amino acids detected were mainly [M+Na]^+^ and [M+2Na-H]^+^ peaks, whereas the other chemicals were mainly [M+Na]^+^ and [M+K]^+^ peaks. At the same laser intensity, glutamic acid was not detected by DHB, and significant background noise was present. In contrast, all 15 compounds present in the plant were detected by the Pt nano pre-coated matrix with a significantly higher S/N. Thus, platinum has a wider detection range and better selectivity.

**Figure 2:**
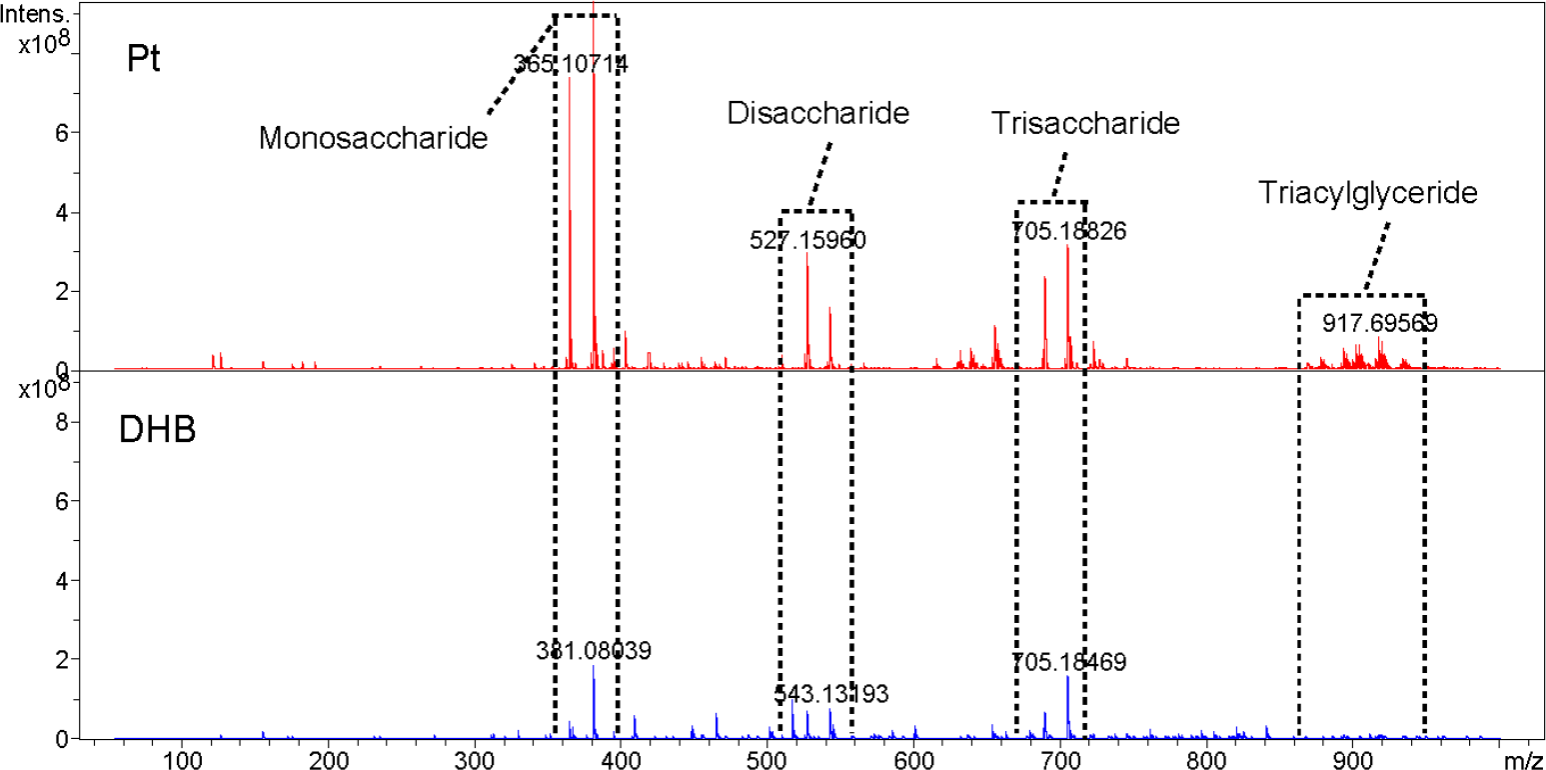
Metabolite detection of soybean extracts by MALDI-FTICR MS using pre-coated Pt and DHB as the matrix, respectively(At 50% laser intensity).

Soybean extracts were examined and contrasted with DHB to confirm the use of Pt nano matrix. **Figure 2** shows the mass spectra, which revealed that the identified compounds were mainly oligosaccharides and lipids. As indicated in **Supplementary Table 2**, the preliminary qualitative identification was based on the accurate molecular weight, isotopic distribution of the high-resolution mass spectra, and reference to relevant literature (Zhang et al., 2018). The platinum pre-coated matrix significantly increased the signal strength under the same mass spectral circumstances, allowing the detection of lipids, while DHB failed to exhibit a triacylglyceride peak. Using sputtered platinum pre-coated matrix, the triacylglyceride signal peak was found at 40% laser intensity, as a comparison, DHB could detect the triacylglyceride peak only at more than 60% laser intensity (**Figure S4**). Thus, our prepared platinum pre-coated matrix requires less laser energy than conventional organic matrixes while detecting more mass spectral peaks.

### MALDI MSI analysis of oligosaccharides and lipids in germinated wheat and corn kernels

**Figure 3:**
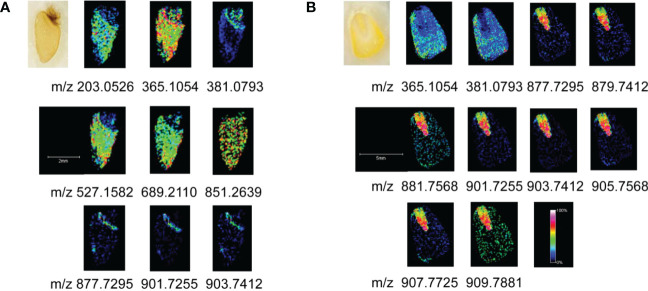
MALDI MSI of oligosaccharides and lipids. **(A)** Germinated wheat; **(B)** corn kernels.

For the wheat seeds to germinate naturally, they were kept at ambient temperature and shielded from light. Wheat seeds undergo several developmental stages after absorbing water, including embryonic enlargement, skin splitting, whitening, bud breaking through the seed coat, rooting, and leaf growth. Following 12 days of germination, when the wheat seeds had developed roots, sprouted leaves, and softened (**Figure S5**), we collected them for MALDI MSI analysis. Monosaccharides (m/z 203.0526), disaccharides (m/z 365.1054), trisaccharides (m/z 527.1582), tetrasaccharides (m/z689.2110), pentasaccharides (m/z851.2639) and triacylglycerides were mainly detected in germinated wheat seeds. We identified oligosaccharides based on the reference pure chemicals and tandem mass spectrometry (see **Figure S6**).The MS data revealed a fairly regular peak pattern, which matched to fragmented oligosaccharides with an interval (m/z = 162) caused by glycosidic cleavages. In the MS/MS of selected precursors, mass differences of hexose residues (162.05 Da) were annotated. Based on the comparison of the reference pure chemicals, MS/MS and highly regular MS peak patterns, these peaks were considered as oligosaccharides with lengths from 2 to 5 hexose residues, known as disaccharides, trisaccharides, tetrasaccharides and pentasaccharides. **Figure 3A** demonstrated the spatial distribution features of the components in the seeds. Oligosaccharides were predominantly present uniformly in the endosperm, with the highest content of disaccharides, whose [M+Na]^+^ peaks were uniformly distributed throughout the endosperm, and the [M+K]^+^ peaks were predominantly present in the outer endosperm and growing shoots, with a higher concentration in the shoots. In addition to disaccharides, the shoots contained fewer oligosaccharides than the endosperm. The embryo was primarily composed of triacylglycerides with m/z values of 877.7295, 879.7412, 901.7255, and 903.7412. During seed germination, amylase converts the starch in the endosperm into glucose and maltose, which supply energy to the embryo and stimulate it’s rooting and germination.

The imaging diagram reveals that the embryo and endosperm are dissimilar and looks perform diverse physiological roles during germination, which is represented in the significant metabolic gap between their separate groups. The great majority of the endosperm in wheat seeds is consisted of starch, which is digested by amylase during germination to form oligosaccharides and then further degraded to produce glucose and maltose (disaccharides). Thus, the high disaccharide content is found in the plant tissue following seed germination. The result is well illustrated by the imaging map, which depicts the high disaccharides concentration in the endosperm.

Fresh sweet corn kernels were fetched for imaging analysis. The results showed that disaccharides and triacylglycerides were mainly present in sweet corn. The primary triacylglycerides were PLL (m/z 877.7295), POL/PLO (m/z 879.7412), POO/PLS (m/z 881.7568), LLL (m/z 901.7255), OLL (m/z 903.7412), OOL (m/z 905.7568), OOO (m/z907.7725) and SOO (m/z 909.7881), in which P is palmitic acid, L is linoleic acid, O is oleic acid and S means stearic acid. **Figure 3B** demonstrated that the distribution of various triacylglycerides differed between the endosperm and embryo of corn, with the embryo containing the majority of triacylglycerides. The disaccharides were mainly distributed in the endosperm, and no other oligosaccharides were detected in the kernels.

## Conclusion

In this study, we proposed a platinum nanomaterial pre-coated matrix as an effective material for various small molecule detection and imaging by MALDI-FTICR MS. The preparation method is simple and can be prepared in batches, which simplifies the experimental steps; high-quality spectra were obtained by optimizing the sputtering time, with good repeatability, greater S/N, and a broader detection range compared to conventional organic matrixes. Using this pre-coated matrix in conjunction with the MALDI MSI technology, high-resolution imaging maps of oligosaccharides and lipids in germinated wheat seeds and fresh corn kernels were acquired. The method also provides basic technical support to study the distribution of oligosaccharides and lipids in plant tissues and will be further utilized to study other plant metabolites in the future.

## Data availability statement

The original contributions presented in the study are included in the article/**Supplementary Material**. Further inquiries can be directed to the corresponding author.

## Author contributions

Y-LS: Writing - original draft, participated in the design of the project, methodology, preliminary research and manuscript revision. S-JZ: Participated in the preliminary research. FY: Participated in preparation of platinum pre-coated matrix. CG: Participated in preliminary research. XX: Writing - original draft, participated in the design of the project, methodology, preliminary research and manuscript revision. All authors contributed to the article and approved the submitted version.
