# Supplementary material for: Prefabricated platinum nanomaterial matrix for MALDI-MS imaging of oligosaccharides and lipids in plant tissues
Source: Front Plant Sci. 2023 Jan 18;14:1105374. doi: 10.3389/fpls.2023.1105374 (PMC9889645; doi:10.3389/fpls.2023.1105374)
Supplement: Supplementary file 1 [file DataSheet_1.pdf]

Supplementary Material

Prefabricated platinum nanomaterial matrix for MALDI-MS imaging of oligosaccharides and lipids in plant tissues

Yu-lin Shen, Si-Jia Zhuang, Fan Yang, Can Gong, Xu Xu*

School of Chemical and Environmental Engineering, Shanghai Institute of Shanghai, China

*** Correspondence:** Xu Xu: xuxu@sit.edu.cn

1 Supplementary Figures

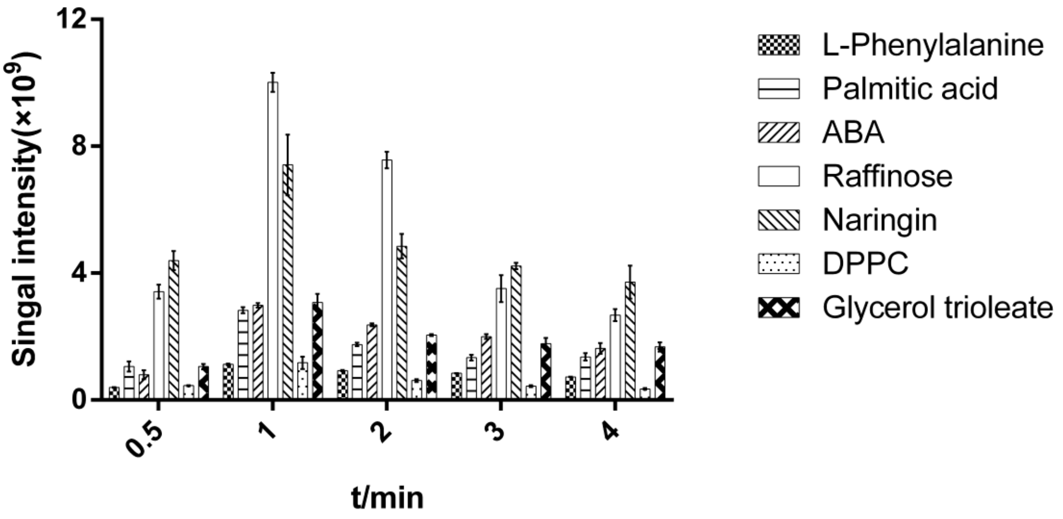

Figure S1. Comparison of standard ion signal intensities at different sputtering times(*i.e.*, 0.5, 1, 2, 3, and 4 min)

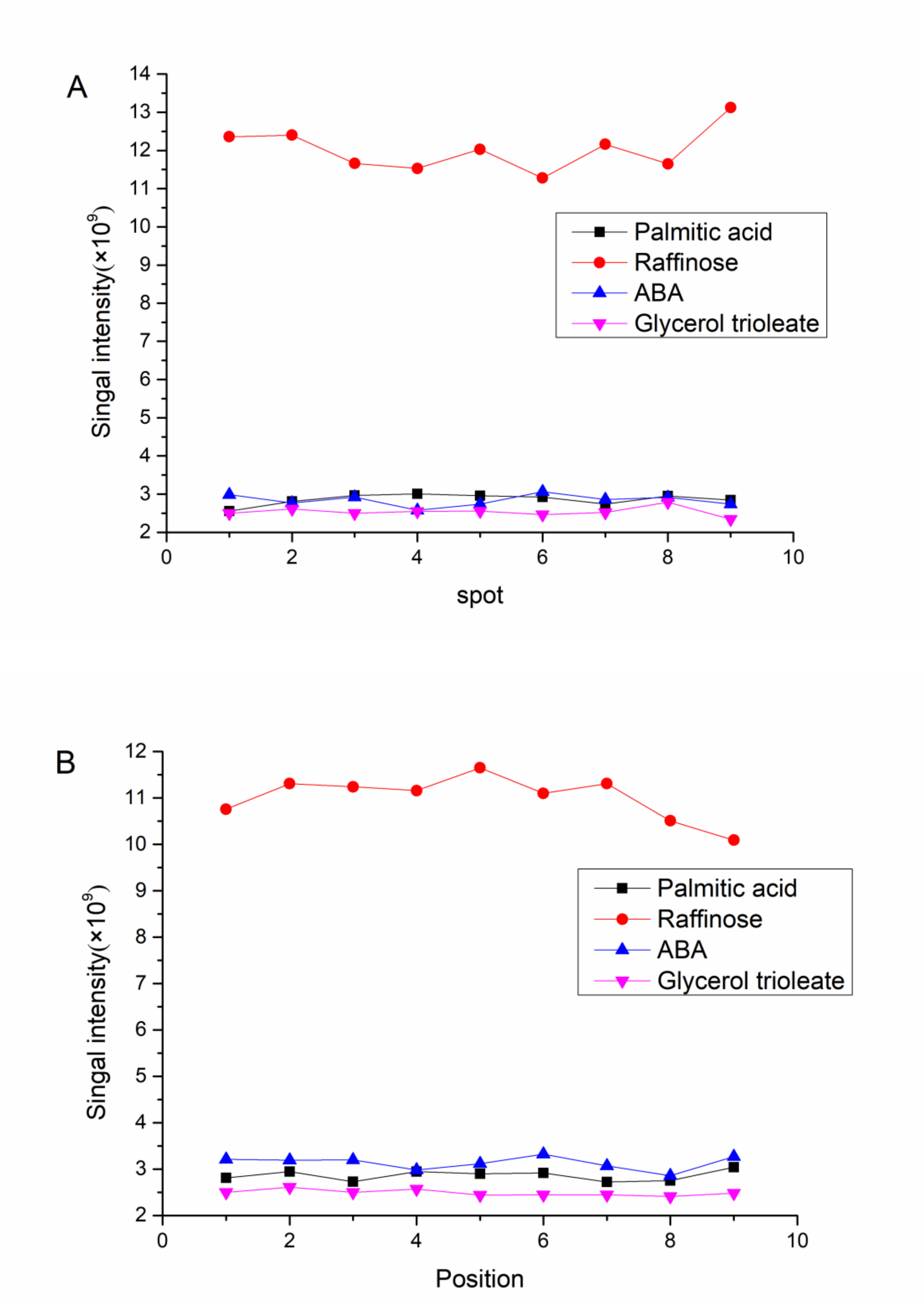

Figure S2. Repeatability test of pre-coated platinum matrix. (A) Repeatability test on 9 different points of palmitic acid, raffinose, abscisic acid and glycerol trioleate；(B) Palmitic acid, raffinose, abscisic acid and glycerol trioleate are carried out in 9 different positions at one point.

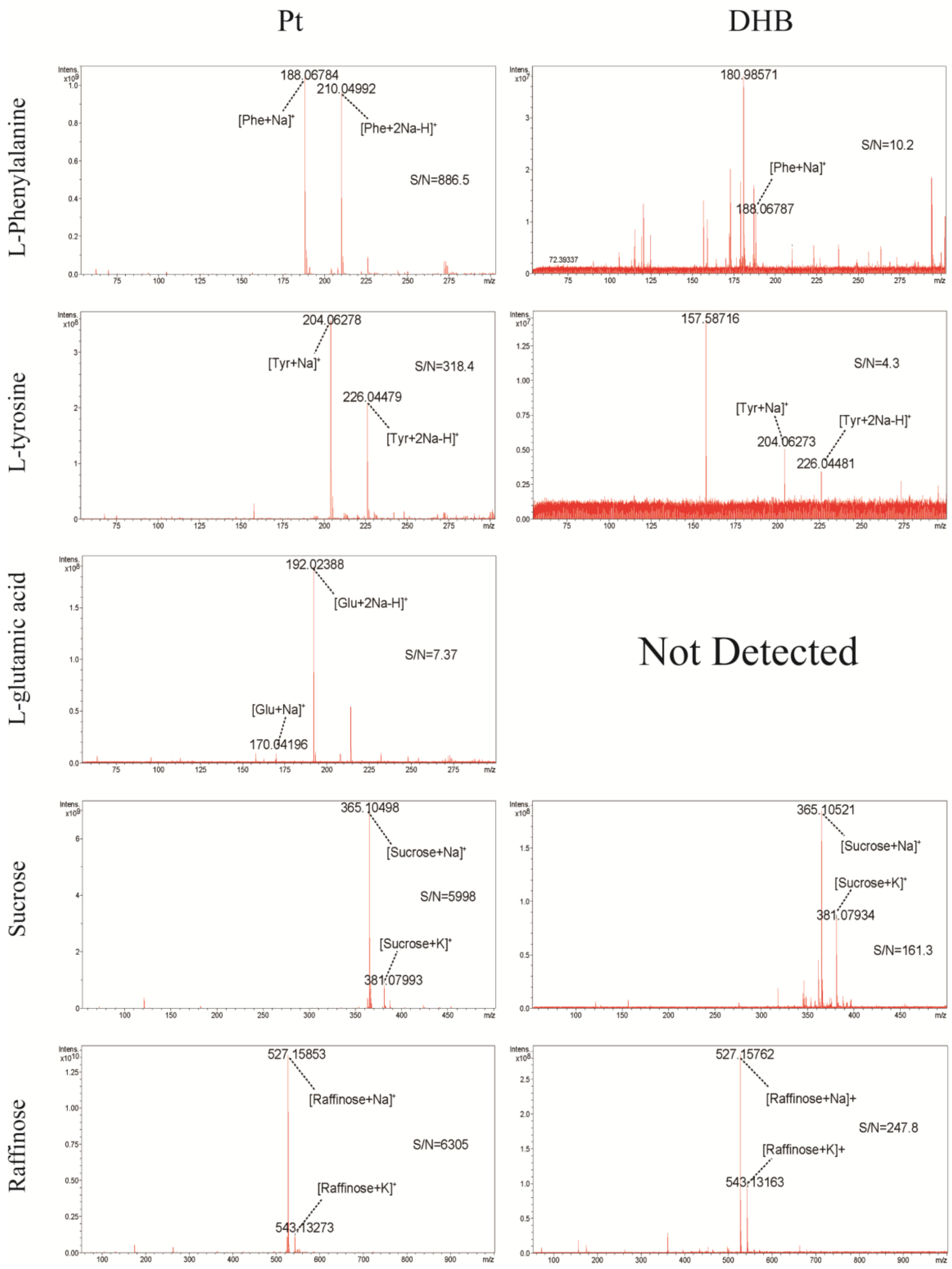

Pt
DHB
L-Phenylalanine
188.06784
210.04992
[Phe+Na]+
[Phe+2Na-H]+
S/N=886.5
180.98571
S/N=10.2
[Phe+Na]+
188.06787
72.39337
L-tyrosine
204.06278
[Tyr+Na]+
S/N=318.4
226.04479
[Tyr+2Na-H]+
157.58716
S/N=4.3
[Tyr+Na]+
[Tyr+2Na-H]+
204.06273
226.04481
L-glutamic acid
192.02388
[Glu+2Na-H]+
S/N=7.37
[Glu+Na]+
170.04196
Not Detected
Sucrose
365.10498
[Sucrose+Na]+
S/N=5998
[Sucrose+K]+
381.07993
365.10521
[Sucrose+Na]+
[Sucrose+K]+
381.07934
S/N=161.3
Raffinose
527.15853
[Raffinose+Na]+
S/N=6305
[Raffinose+K]+
543.13273
527.15762
[Raffinose+Na]+
S/N=247.8
[Raffinose+K]+
543.13163
Intens.
m/z

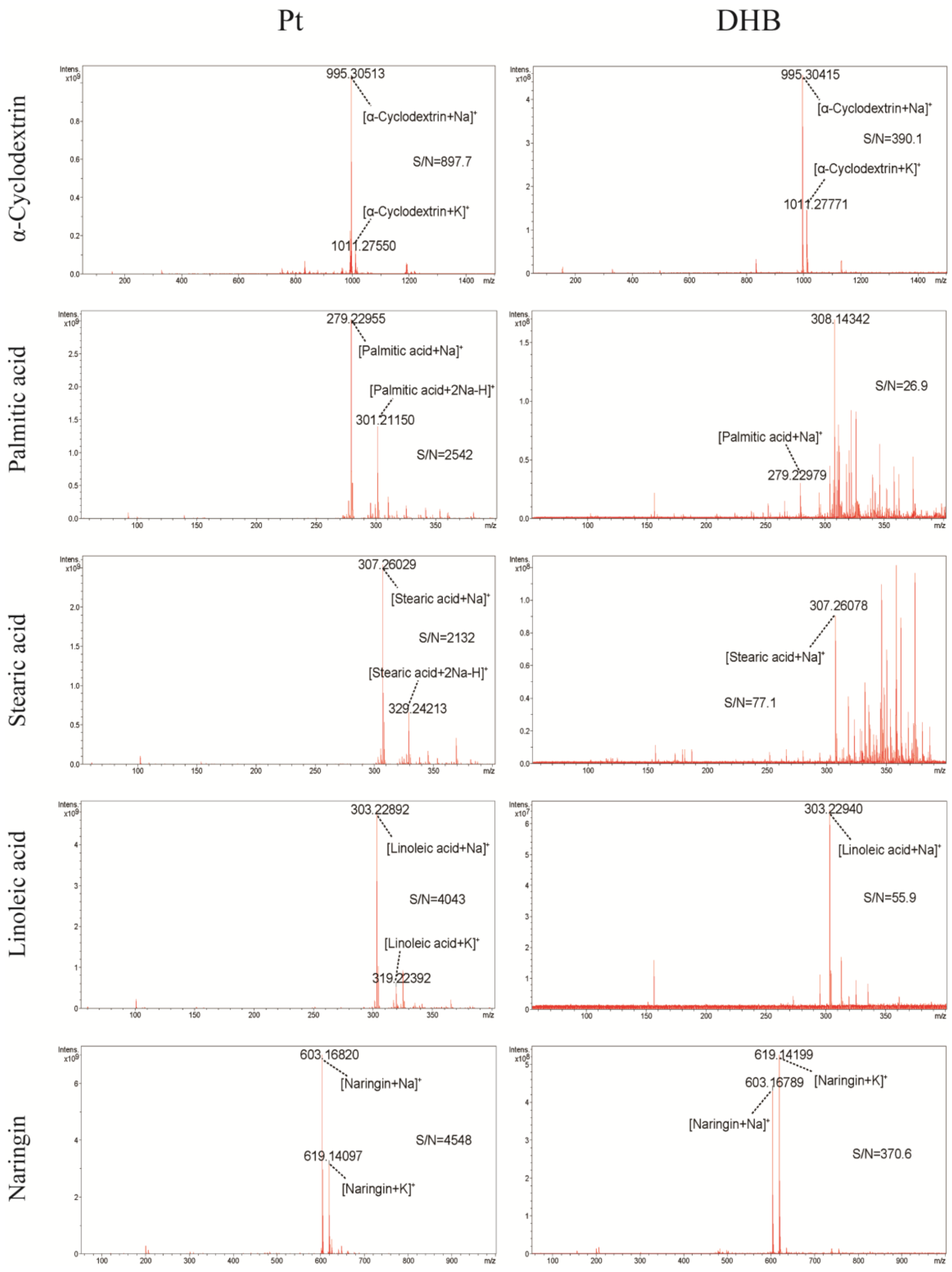

Pt
DHB
α-Cyclodextrin
995.30513
[α-Cyclodextrin+Na]+
S/N=897.7
[α-Cyclodextrin+K]+
1011.27550
995.30415
[α-Cyclodextrin+Na]+
S/N=390.1
[α-Cyclodextrin+K]+
1011.27771
Palmitic acid
279.22955
[Palmitic acid+Na]+
[Palmitic acid+2Na-H]+
301.21150
S/N=2542
308.14342
S/N=26.9
[Palmitic acid+Na]+
279.22979
Stearic acid
307.26029
[Stearic acid+Na]+
S/N=2132
[Stearic acid+2Na-H]+
329.24213
307.26078
[Stearic acid+Na]+
S/N=77.1
Linoleic acid
303.22892
[Linoleic acid+Na]+
S/N=4043
[Linoleic acid+K]+
319.22392
303.22940
[Linoleic acid+Na]+
S/N=55.9
Naringin
603.16820
[Naringin+Na]+
S/N=4548
619.14097
[Naringin+K]+
619.14199
603.16789
[Naringin+K]+
[Naringin+Na]+
S/N=370.6

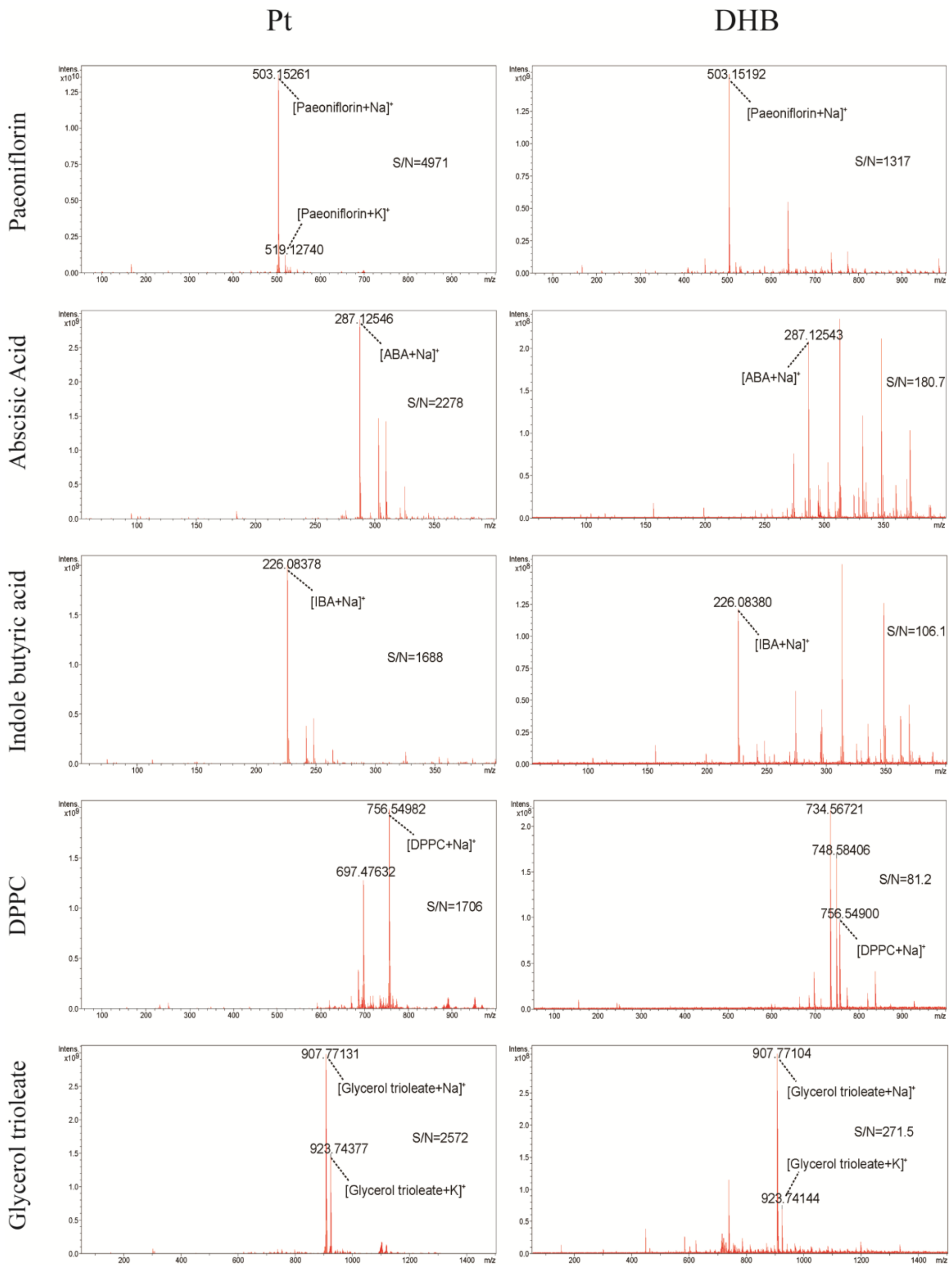

Figure S3. Comparison of the ability of pre-coated platinum and DHB matrix to detect small molecule compounds under the same mass spectrometry conditions

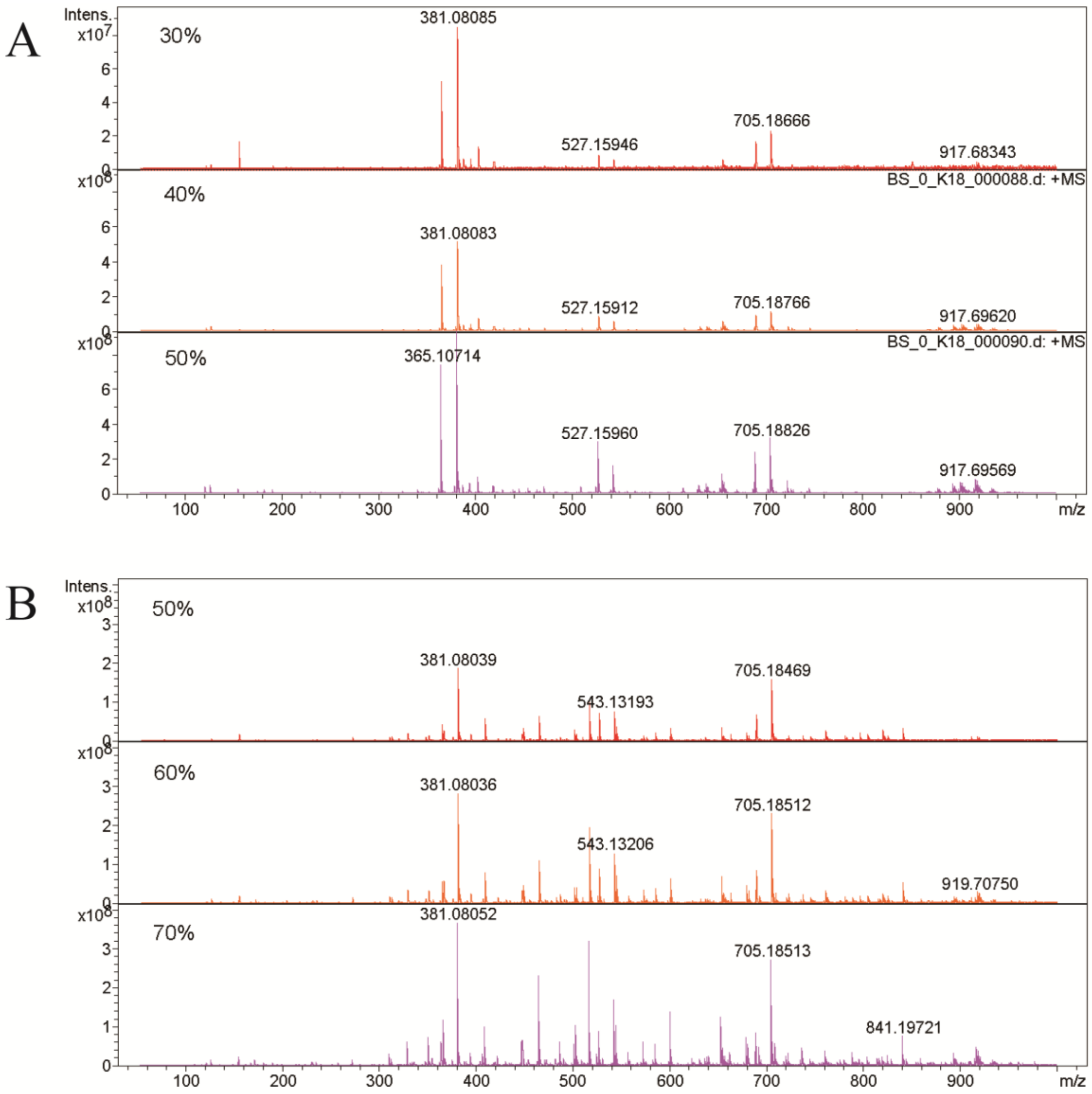

Figure S4. Mass spectra of soybean extracts at different laser intensities. (A) Detection with platinum pre-coated matrix at 30%, 40%, 50% laser intensity, and (B)Detection with DHB pre-coated matrix at 50%, 60%, and 70% laser intensity.Triacylglyceride peaks were detected at 40% laser intensity on platinum pre-coated matrix, as a comparison, DHB could detect the triacylglyceride peak only at more than 60% laser intensity.

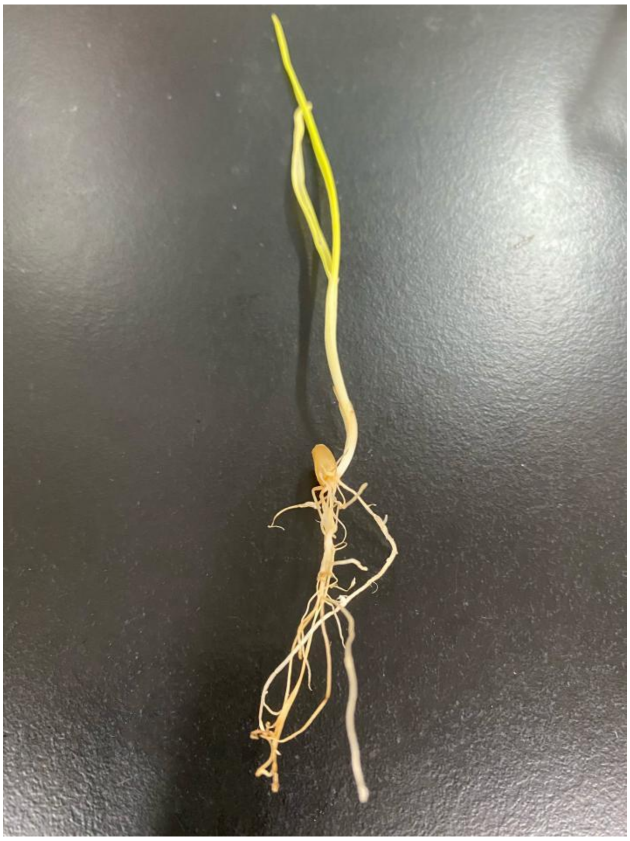

Figure S5. Photo of germinated wheat

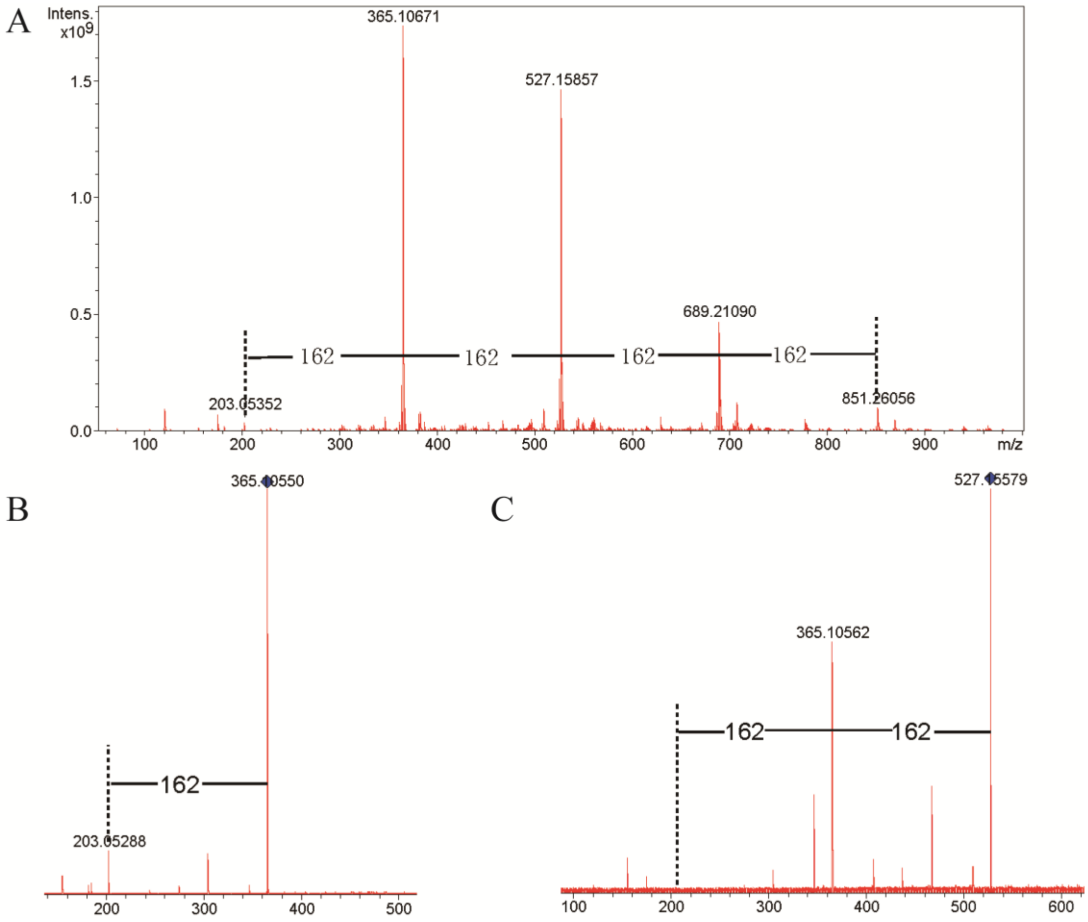

Figure S6.(A)Mass spectra of oligosaccharides in germinated wheat tissue sections, (B)Tandem mass spectra of disaccharides, (C)Tandem mass spectra of trisaccharides.

2 Supplementary Tables

Table S1: Platinum pre-coated matrix for detection of various small molecules

Compound	molecular Formula	Ionic Species	Experimental	Calculated	Δm(ppm)
L-Phenylalanine	$C_9H_{11}NO_2$	M+Na	188.0678	188.0682	-2.13
L-tyrosine	$C_9H_{11}NO_3$	M+Na	204.0627	204.0631	-1.96
L-glutamic acid	$C_5H_9NO_4$	M+Na	170.0419	170.0423	-2.35
Sucrose	$C_{12}H_{22}O_{11}$	M+Na	365.1049	365.1054	-1.37
Raffinose	$C_{18}H_{32}O_{16}$	M+Na	527.1585	527.1582	0.57
α -Cyclodextrin	$C_{36}H_{60}O_{30}$	M+Na	995.3051	995.3061	-1.00
Palmitic acid	$C_{16}H_{32}O_2$	M+Na	279.2295	279.2294	0.36
Stearic acid	$C_{18}H_{36}O_2$	M+Na	307.2602	307.2607	-1.63
Linoleic acid	$C_{18}H_{32}O_2$	M+Na	303.2289	303.2294	-1.65
Naringin	$C_{27}H_{32}O_{14}$	M+Na	603.1682	603.1684	-0.33
Paeoniflorin	$C_{23}H_{28}O_{11}$	M+Na	503.1526	503.1523	0.60
Abscisic Acid	$C_{15}H_{20}O_4$	M+Na	287.1254	287.1253	0.35
Indole butyric acid	$C_{12}H_{13}NO_2$	M+Na	226.0837	226.0838	-0.44
DPPC	$C_{40}H_{80}NO_8P$	M+Na	756.5498	756.5513	-1.98
Glycerol trioleate	$C_{57}H_{104}O_6$	M+Na	907.7713	907.7725	-1.32

Table S2: Compounds in soybean extract detected in positive ion mode using Platinum nanomaterials as pre-coated matrix

Compound Identification	molecular Formula	Ionic Species	Experimental	Calculated	Δm(ppm)
Disaccharide	$C_{12}H_{22}O_{11}$	M+Na	365.1055	365.1054	0.27
		M+K	381.0798	381.0793	1.31
Trisaccharide	$C_{18}H_{32}O_{16}$	M+Na	527.1576	527.1582	-1.14
		M+K	543.1319	543.1321	-0.37
Tetrasaccharide	$C_{24}H_{42}O_{21}$	M+Na	689.2101	689.2110	-1.31
		M+K	705.1830	705.1850	-2.84
PC(34:2)	$C_{42}H_{80}NO_8P$	M+K	796.5252	796.5253	-0.13
PC(36:4)	$C_{44}H_{80}NO_8P$	M+K	820.5278	820.5253	3.05
PPL	$C_{53}H_{98}O_6$	M+Na	853.7231	853.7255	-2.81
		M+K	869.6972	869.6994	-2.53
PPO	$C_{53}H_{100}O_6$	M+Na	855.7423	855.7412	1.29
		M+K	871.7138	871.7151	-1.49
PLL	$C_{55}H_{98}O_6$	M+Na	877.7250	877.7295	-5.13
		M+K	893.6956	893.6994	-4.25
POL	$C_{55}H_{100}O_6$	M+Na	879.7400	879.7412	-1.36
		M+K	895.7119	895.7151	-3.57
POO	$C_{55}H_{102}O_6$	M+Na	881.7542	881.7568	-2.95
		M+K	897.7275	897.7307	-3.56
PLS	$C_{55}H_{102}O_6$	M+Na	881.7542	881.7568	-2.95
		M+K	897.7275	897.7307	-3.56
LnLLn	$C_{57}H_{94}O_6$	M+K	913.6682	913.6681	-0.11
LLLn	$C_{57}H_{96}O_6$	M+Na	899.7072	899.7099	-3.00
LLL	$C_{57}H_{98}O_6$	M+Na	901.7241	901.7255	-1.55
		M+K	917.7020	917.6994	2.83
OLLn	$C_{57}H_{98}O_6$	M+Na	901.7241	901.7255	-1.55
		M+K	917.7020	917.6994	2.83
OLL	$C_{57}H_{100}O_6$	M+Na	903.7383	903.7412	-3.21
SLLn	$C_{57}H_{100}O_6$	M+Na	903.7383	903.7412	-3.21
OOL	$C_{57}H_{102}O_6$	M+Na	905.7530	905.7568	-4.20
		M+K	921.7297	921.7307	-1.08
SLL	$C_{57}H_{102}O_6$	M+Na	905.7530	905.7568	-4.20
		M+K	921.7297	921.7307	-1.08
OOO	$C_{57}H_{104}O_6$	M+Na	907.7675	907.7725	-5.51
SOL	$C_{57}H_{104}O_6$	M+Na	907.7675	907.7725	-5.51
PLG	$C_{57}H_{104}O_6$	M+Na	907.7675	907.7725	-5.51
SOO	$C_{57}H_{106}O_6$	M+K	925.7605	925.7620	-1.62
PLA	$C_{57}H_{106}O_6$	M+K	925.7605	925.7620	-1.62

P: Palmitic acid; L:Linoleic acid; O: Oleic acid; S: Stearic acid; Ln: Linolenic acid; A: Arachidic acid
